# The Effect of “Motivational Interviewing” and “Information, Motivation, and Behavioral Skills Model” Counseling Interventions on the Choice of Delivery Mode in Pregnant Women Using Face-to-Face Training vs. Mobile App: A Randomized Controlled Trial

**DOI:** 10.1155/2024/3071183

**Published:** 2024-09-30

**Authors:** Mahboubeh Shirzad, Elham Shakibazadeh, Payam Sheikhatari, Abbas Rahimi Foroushani, Hamid Poursharifi

**Affiliations:** ^1^ Department of Health, Behavior & Society Bloomberg School of Public Health Johns Hopkins University, Baltimore, MD, USA; ^2^ Department of Health Education and Health Promotion School of Public Health Tehran University of Medical Sciences, Tehran, Iran; ^3^ School of Community Health and Policy Prevention Sciences Research Center Morgan State University, Baltimore, MD, USA; ^4^ Department of Epidemiology and Biostatistics School of Public Health Tehran University of Medical Sciences, Tehran, Iran; ^5^ Department of Psychology University of Social Welfare and Rehabilitation Sciences, Tehran, Iran

## Abstract

**Objective:**

To investigate the impact of counseling interventions, using face-to-face training vs. mobile app for choosing mode of delivery.

**Design:**

A four-armed, randomized, controlled parallel-design trial. *Setting*. Ebnesina Private Hospital in Tehran, Iran. *Population*. Pregnant women, between 24 and 32 weeks of gestation (*n* = 120).

**Methods:**

Pregnant women were randomly assigned in three psycho-educational intervention groups: (1) motivational interviewing via face-to-face training, (2) information, motivation, and behavioral skills model via face-to-face training, (3) the same model via a mobile application, and (4) usual antenatal care (control group). To assess the face-to-face and mobile app training method on women's self-efficacy and intention in choosing a mode of delivery. *Main Outcome Measures*. Mode of delivery (Cesarean section).

**Results:**

While all three intervention groups showed significant increases in women's self-efficacy and intentions to choose vaginal delivery, the increase was particularly noticeable among those using mobile applications: Before the intervention, self-efficacy and intention Mean ± SD were 77.1 ± 38.6 (CI-95%: [62.72, 91.60]) and 1.10 ± 0.305 (CI-95%: [0.99, 1.21]), respectively. After the intervention, these scores increased to 99.7 ± 30.7 (CI-95%: [88.27, 111.20]) for self-efficacy and 1.70 ± 0.466 (CI-95%: [1.53, 1.87]) for intention. Although 56.7% of women in the intervention groups expressed a preference for vaginal delivery, only 37.5% ultimately pursued this birthing method.

**Conclusions:**

Brief psycho-educational interventions, particularly technology-driven interventions (mobile apps) can increase the likelihood of women choosing vaginal delivery. To enhance the effectiveness of such interventions, they can be conducted in conjunction with interventions for doctors and healthcare providers. This trial is registered with IRCT20151208025431N7.

## 1. Introduction

The increasing rate of unnecessary Cesarean Section (CS) is a challenging health issue around the globe [1]. Iran has one of the highest rates of CS among Middle Eastern countries, ranging from 26% to 60% in private hospitals, often without justifiable medical indications [2].

Although many factors contribute to the high rate of CS, including economic, organizational, social, and cultural factors [3, 4], women's preferences play a particularly important role in decision-making about mode of delivery [5–7]. This process can be affected by factors such as fear of pain [8–10], concerns about genital modifications, misinformation [11–14], and the convenience of CS [12]. Addressing these underlying factors through comprehensive education programs for both women and healthcare providers could significantly impact women's choices and ultimately contribute to reducing CS rates. Therefore, educating is essential for reducing CS rates.

Iranian health system has implemented the “Health Sector Evolution Policy” [15] in 2014 to combat unnecessary CS. This initiative includes measures such as free vaginal delivery (VD) services in public hospitals, mother-friendly facilities, standardized childbirth protocols, prenatal classes, improved labor privacy and infrastructure, enhanced birthing facilities, promotion of water births, and financial incentives for doctors to encourage VDs in public Hospitals [16]. Despite these efforts, the CS rate remains notably high [16].

Various psycho-educational approaches exist for behavior changing. Motivational interviewing (MI), a patient-centered counseling approach, is a powerful method for changing unhealthy behaviors [17], especially among individuals who are not yet ready to change [18]. It involves clarifying patient goals for changing unhealthy behaviors, exploring obstacles, and making commitments to change [18]. Another approach is the Information-Motivation-Behavioral Skills (IMB) model, a simple and adaptable model for complex health behaviors. It provides information and motivation, and enhances necessary skills for behavior change [19]. Information includes fundamental knowledge about the condition and effective strategies, motivation involves personal attitudes and social support, and behavioral skills include tools and strategies for adherence, like social support and self-regulation techniques [20–23].

Using nonclinical interventions such as psycho-educational interventions is recommended by WHO [24] as an effective strategy [25, 26] which can assist pregnant women in making informed decision about their mode of delivery. Nonclinical interventions effectively reduced the fear of childbirth among pregnant women [27].

Delivering educational content, especially for nonclinical interventions using technology has become a simple and inexpensive channel [28]. This is supported by research demonstrating its effectiveness in various areas, including smoking cessation [29], medication adherence [30], and blood pressure management [31]. To support the importance of delivering educational content through technology, especially for nonclinical interventions, several studies highlight its effectiveness in various health-related domains. For example, the results of a randomized controlled trial conducted by Juyani et al., 2024 showed that a mobile-based educational intervention had a significant effect on the preventive behaviors of STIs in women. [32] Another randomized controlled trial showed improvements on cervical cancer prevention behaviors through mobile-based educational intervention. [33] These findings support the potential benefits of using mobile apps as an effective alternative to face-to-face training. This research further expands on this theme by comparing the effect of motivational interviewing and information-motivation-behavioral skills delivered via mobile app and face-to-face training on pregnant women's choice of delivery mode.

## 2. Methods

### 2.1. Study Design

A four-armed, randomized, controlled parallel-design trial was conducted to compare the effects of MI and IMB model counseling interventions on the choice of delivery mode in pregnant women. The interventions were delivered through face-to-face sessions and a mobile application, named: “The Easy Birth” within 6 months

### 2.2. Protocol and Registration

This research was conducted in accordance with the Declaration of Helsinki and relevant guidelines and regulations. The study protocol has been published in the BioMed Central (BMC) Trials journal [34].

### 2.3. Participants (Inclusion and Exclusion Criteria) and Settings

**Figure 1:**
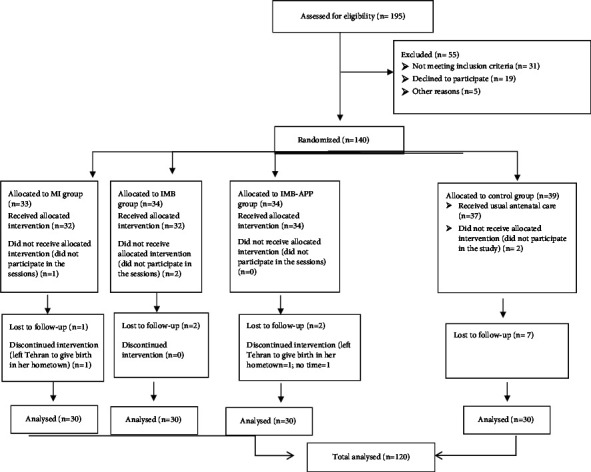
CONSORT flow diagram.

We recruited 120 pregnant women who were referred to Ebnesina Private Hospital in Tehran, Iran, from 2019 to 2021. The inclusion criteria were as follows: being literate (i.e., being able to read and write), having a gestational age between 24 and 32 weeks at the time of recruitment, speaking Farsi, experiencing no complications in the current pregnancy, having no indications for CS, and having sufficient time to participate in the intervention sessions. Women who experienced medical complications during the study and/or had preterm labor were excluded (Figure 1).

### 2.4. Interventions

Educational content tailored to our groups' needs was developed based on the findings of a qualitative evidence synthesis, a quantitative systematic review, and a meta-analysis [5], as well as a qualitative study conducted in Iran [15] to identify factors that affected women's preferences in choosing their mode of delivery.

To compare the effectiveness of various psycho-education interventions delivered through face-to-face training and a mobile app, we enrolled pregnant women into three intervention groups and one control group. Our intervention groups included: (1) MI with face-to face training; (2) IMB model with face-to-face training; and (3) IMB model using a mobile app (IMB-App). Three-session brief interventions were designed and delivered to pregnant women during their prenatal care appointments. The MI and IMB face-to-face sessions were conducted by a midwife and a behavioral specialist who were trained by a health psychologist (H. P.). The IMB-App was designed to deliver intervention content to pregnant women, similar to our face-to-face intervention content. The first session was accessible to the pregnant women at the time of recruitment. Other sessions were made available after the pregnant women completed the previous session. There was no time limitation in the IMB-App group, and they had access to the application information throughout the intervention.

#### 2.4.1. MI Intervention Group (Face-to-Face)

After introducing MI techniques, pregnant women were interviewed face-to-face during three 45–60 minute sessions. In this intervention group, we assisted pregnant women in exploring and resolving ambivalence regarding their choice of delivery mode and in building their intrinsic motivation. A series of open-ended questions were asked to pregnant women to understand their thoughts on CS and VD. For example, they were asked, “Tell me what you think about CS?” What encourages you to choose CS or VD? To support and encourage pregnant women in further exploring their decision-making process, we utilized affirming and reflective listening, which is a crucial component of MI. For example, a pregnant woman said: “I'm really scared of having a vaginal delivery. I can't even think about it”. Counselor said: “I understand that you're scared. Vaginal delivery can be painful, but it is important to know that there are many ways to reduce pain during vaginal delivery, and we can work together to find the best options for you” (Table S1).

#### 2.4.2. IMB Intervention Group (Face-to-Face)

In three 45–60 minute sessions, pregnant women received information and behavioral skills training on how to decide on their mode of delivery by assessing the strengths and weaknesses of each option. During these sessions, pregnant women received information on complications related to CS for both the mother and infant, as well as the outcomes of unnecessary CS. They were motivated through providing personal feedback, reiterative questioning, affirmation, and reflective listening. Additionally, they were encouraged by defining realistic goals for choosing CS (considering their body shape, fear, and infant health). Finally, they were provided with specific behavioral skills training on how to control and modify their behavior (Table S1).

#### 2.4.3. IMB Intervention Group (Mobile App)

A mobile application was designed according to the IMB model (The Easy-Birth mobile app was designed for the purpose of this study [35]). The same IMB intervention content was used and delivered in three parallel sessions via the IMB-App. To integrate self-efficacy into the mobile app, we considered the following strategies: (1) personalized feedback; (2) educational content and skills training; (3) social support and interactions; (4) motivational messaging; and (5) interactive and engaging design. To enhance the participants' intention in our mobile app, we implemented the following strategies: (1) clear and compelling value proposition; (2) behavioral reminders; and (3) providing feedback.

We implemented the following features based on the strategies provided: Our app was designed user-centered and responsive; we designed the app with a clean, simple interface that allowed the participants to easily find and use different features. We ensured the app worked smoothly on various devices and screen sizes. We provided easy access and educational content and information. We provided clear and compelling value proposition; we highlight key benefits and success stories during the onboarding process to show users the value of the app right from the start. Offline mode allowed the participants to access certain features and log data even without Internet connection. By incorporating these features, the app could better support the participants in achieving their health goals, thereby improving overall engagement and effectiveness.

We tested the app usability at early prototyping stage to identify major usability issues early on, and the post-launch stage to gather ongoing user feedback to make continuous improvements. We used the “Think-Aloud Protocol” to test the app's usability. The participants verbalized their thoughts while using the app. This protocol provided insights into the participants' thought processes and identified usability issues.

We implemented logging mechanisms to record each time a participant opened the app, navigated through different sections, and completed specific tasks or modules to monitor the compliance with the app.

Session Duration: Measure the duration of each session to understand how long users engage with the app per session.

The software was installed on the mobile phones of the pregnant women in the IMB-App group, and individual technical assistance was offered to them. Women worked with the application in the presence of one of the researchers to resolve existing problems. The strategies for improving adherence included setting reminders at defined intervals in the form of pop-up messages. Data collected on the server was analyzed to monitor the women's adherence. In addition to the application, a server was designed to collect user activities. Data collected included the duration of app usage, the sections accessed by the user, and the time spent in each section. Every time the users' mobile phones were connected to the Internet, the data was uploaded and saved on the server. These data were used as proxies to measure adherence to the intervention.

### 2.5. Outcomes

Our study aimed to:

1. Determine and compare the effects of MI, IMB, and IMB-App on the mode of delivery in the target and control groups before and after the interventions;
2. Determine and compare the effects of MI, IMB, and IMB-App on the intention to perform CS in women in the target and control groups before and after the interventions; and
3. Determine and compare the effects of MI, IMB, and IMB-App on women's self-efficacy in both target and control groups before and after the interventions.

### 2.6. Sample Size

One hundred and twenty pregnant women were included in the study with a Type I error (alpha) of 0.05 and a test power of 80% to detect a minimum difference of at least 20% in the rate of CS due to the intervention. Considering that the rate of the CS in the control group is 50%, which was reported as 47.9% [36], the sample size in each group was determined with 95% confidence.

### 2.7. Randomization

After the initial assessment and completion of the baseline measurement, pregnant women were randomly assigned to four groups. Pregnant women were recruited based on the order of clinic registration. Each woman on the list was assigned a consecutive research identification number in order of their registration with the clinic. The allocation was performed by a clinical nurse who was not aware of the study objectives.

### 2.8. Measures

We used a valid and reliable questionnaire consisting of 17 items about self-efficacy and two items about the intention to choose the mode of delivery [37, 38]. Moreover, demographic information including age, income, educational level, and employment status of both pregnant women and their partners, number of births, pregnancies, current gestational age (at recruitment), number of live children, history of infertility, history of illness, date of birth, previous participation in birth preparation classes, and preferred delivery mode were collected.

After obtaining consent from pregnant women, we provided them with the questionnaire to complete. For the IMB-App group, we also installed the mobile application on their cell phones. We followed the women until delivery to understand their chosen mode of delivery. During the interventions, if a participant was unwilling to continue, we replaced them with another individual.

### 2.9. Statistical Methods

We used the Shapiro–Wilk Test for assessing the normality of the data, and the *p* value was greater than 0.05, which indicated that the data did not significantly deviate from a normal distribution. Data were analyzed using descriptive statistics (mean, frequency, and standard deviation), inferential statistics such as a two-independent *t*-test, paired *t*-test, and Chi-squared test to examine the factors affecting women's choice of mode of delivery to assess the combined impact of variables on the likelihood of choosing CS. The significance level of the test was set at less than 0.05.

### 2.10. Patient Consent and Confidentiality

All participants were informed about the study and its purposes and were assured that all information collected would remain confidential. Each participant signed an informed consent form

## 3. Results

Five participants in the intervention groups and seven in the control group were lost to follow-up. For more details, please refer to Figure 1. Most pregnant women (57.5%) were between 18 and 30 years old and nulliparous and 70.0% of them being employed. No significant differences were found between the groups (Table 1).

After implementing the intervention, a paired samples *t*-test revealed a significant improvement in self-efficacy, and intention among pregnant women compared to the control group. It showed that the Mean ± SD self-efficacy among pregnant women in the IMB-App group was 77.1 ± 38.6 (CI-95%: [62.72, 91.60]) before the intervention and 99.7 ± 30.7 (CI-95%: [88.27, 111.20]) after the intervention. In the IMB group, the mean self-efficacy was 86.8 ± 24.5 (CI-95%: [77.70, 96.03]) before and 103.2 ± 24.9 (CI-95%: [93.92, 112.55]) after the intervention. In the MI group, it was 66.1 ± 18.2 (CI-95%: [59.38, 73.49]) before and 80.5 ± 22.7 (CI-95%: [71.97, 89.16]) after the intervention. However, there were no significant differences before (81.77 ± 40.82 (CI-95%: [64.80, 96.60])) and after the interventions (84.33 ± 3.67(CI-95%: [70.85, 97.32])) in the control group (*p* < 0.47) (Table 2). Moreover, women's intention to undergo VD increased significantly after the intervention (*p* < 0.001), with a greater improvement observed among women in the IMB-App group compared to the other intervention groups 1.10 ± 0.305 (CI-95%:[0.99, 1.21]) and 1.70 ± 0.466 (CI-95%: [1.53, 1.87]). For more details, please refer to Table 2.

An independent-samples *t*-test was conducted to compare self-efficacy in CS and VD before and after the interventions. There was a significant difference in CS (66.30 ± 04.71) and VD (89.04 ± 34.28); *t* = 2.978, *p*=0.004 before the intervention, and a significant difference in CS (86.41 ± 30.62) and VD (101.09 ± 27.53); *t* = 2.637, *p*=0.009 after the intervention (Table 2).

Our analysis also showed that although 56.7% of women preferred VD, only 37.5% underwent VD, while 62.5% underwent CS indicating differences between women's preferred and actual mode of delivery (Table 3).

We also calculated the number of women who underwent CS and VD. After the intervention, the number of women who preferred VD increased overall compared to the control group. However, the women in the IMB-App group were more likely to choose VD than the women in the MI and IMB face-to-face intervention groups (Table 4)

## 4. Discussion

### 4.1. Main Findings and Interpretation

In this randomized controlled trial study that examined the effect of MI and IMB model counseling interventions on the choice of delivery mode in pregnant women using face-to-face sessions and a mobile app, we found that psycho-educational interventions can help pregnant women feel more confident and motivated to choose VD over CS. Although psycho-education interventions are recommended by the WHO as a valuable approach to decreasing the trend of CS [24], we believe that this type of intervention, due to the influence of other potential factors leading pregnant women to undergo CS (e.g., doctors' recommendations during childbirth), can be used as a supplementary method for educating pregnant women on selecting the appropriate mode of delivery. Policy changes, environmental factors, hospital settings, and services should be considered when implementing multilevel interventions to decrease the rate of CS in Iran.

Self-efficacy is a crucial factor in enhancing women's ability to cope with labor, which means those with higher self-efficacy can better cope with labor. It implies that women with higher self-efficacy are better able to manage labor pain during childbirth compared to those with lower self-efficacy. Our results indicated that women with higher self-efficacy tended to choose VD more frequently, which aligns with the findings of Dilks et al. [39] and Taheri et al. [40]. Thus, improvements in self-efficacy can empower women to choose the appropriate mode of delivery based on their medical conditions.

Using a mobile application to deliver educational content led to significantly greater improvements in self-efficacy and intention among pregnant women in our study. Mobile application–driven interventions are widely used as a channel for delivering health education. Nowadays, most people have access to mobile phones; and women make up about 45% of mobile users in Iran [41]. Therefore, health information can reach this target group widely [42]. Via mobile app technology, we can easily deliver health content to our target group to provide them with health information [43]. Therefore, using mobile devices to encourage pregnant women to make decision about their mode of delivery and motivate them to adopt healthy behaviors is an effective tool [44]. We are now aware of the effectiveness of online education compared to traditional health education training [45]. The ongoing transition from traditional education to virtual education as a novel educational approach can facilitate simple and inexpensive access to educational resources and services regardless of time and place. A new phase of evolution in education, known as “online education,” enables users to access information easily [46]. The value of this type of education has been highlighted during the COVID-19 pandemic. Women at risk of pregnancy-related complications need to receive their education through accessible tools via trusted channels.

This study demonstrated that psycho-educational interventions could significantly boost pregnant women's self-efficacy and intention to choose vaginal delivery, particularly through mobile app–based interventions. This suggests that incorporating digital health tools can effectively support patients' decision-making processes. To further improve outcomes, it is recommended to complement patient-focused interventions with programs aimed at educating and supporting healthcare providers. This dual approach can address any potential provider biases and ensure consistent, supportive counseling for pregnant women considering their delivery options.

Moreover, healthcare policymakers and practitioners should consider integrating mobile app-based educational tools into standard antenatal care. These tools can help bridge gaps in patient education, particularly in regions with limited access to face-to-face counseling services. By implementing these insights into clinical practice, healthcare providers can enhance the decision-making experience for pregnant women, potentially reducing the rates of unnecessary CS and promoting safer, more informed delivery choices.

### 4.2. Strengths and Limitations

Our study utilized a rigorous randomized controlled trial design to evaluate our intervention in a nonclinical setting. We delivered the intervention to participants in person and through a mobile app. The main limitation of our study was the small sample size. To assess the feasibility of implementing this intervention in women's healthcare programs, we need to conduct studies on larger populations. Additionally, we only recruited participants from private hospitals, as elective CS are not allowed in public hospitals in Iran.

## 5. Conclusion

Implementation frameworks related to psycho-education interventions need further study to identify barriers and facilitators of implementing such strategies. We conducted our study in a private hospital; however, future studies are needed to assess the effectiveness of similar interventions in public health centers with a larger number of participants and to measure more outcomes, such as the APGAR score of newborn babies. We suggest conducting additional studies to evaluate the effects of comprehensive interventions aimed at women, healthcare professionals, and healthcare systems. Identifying the reasons behind the high rates of CS within various specific contexts could inform the design of more cost-effective educational and motivation enhancement interventions.

To enhance the effectiveness of healthcare services at the local, regional, and national levels, we can introduce brief interventions for pregnant women in conjunction with interventions for doctors and healthcare providers. We could also use a mobile app to assess the effects of these interventions and related policies based on the local context. Additionally, we can use simple, cost-effective, and culturally sensitive brief interventions customized for women's prenatal care appointments to assist them in making informed decisions regarding their mode of delivery. To achieve this, we should adopt a variety of implementation strategies to tailor brief interventions to the needs of individuals from diverse backgrounds and various prenatal clinics.

## Figures and Tables

**Table 1:** Demographic information of the participant women (*n* = 120).

Demographic information		MI		IMB		IMB-App		Control group		Total		Chi-squared (*χ*^2^)
		*N*	%	*N*	%	*N*	%	*N*	%	*N*	%	*p* value
Age	18–30 years	18	60.0	16	53.3	13	43.3	10	33.3	56	57.5	0.178
	>30 years	12	40	14	46.7	17	56.7	20	66.7	63	52.5	

Parity	Nulliparous	14	46.7	20	66.7	21	70.0	14	46.7	69	57.5	0.242
	Primiparous or multiparous	16	53.3	10	33.3	9	30.0	16	53.3	51	42.5	

Previous deliveries	1	14	46.7	23	76.7	22	73.3	15	50.0	74	61.7	0.106
	2	10	33.3	6	20.0	5	16.7	11	36.7	32	26.7	
	>2	6	20.0	1	3.3	3	10.0	4	13.3	14	11.7	

Number of children	0	12	40.0	18	60.0	24	80.0	16	53.3	70	57.3	0.086
	1	12	40.0	9	30.0	4	13.3	11	36.7	36	30.0	
	>1	6	20.0	3	10.0	2	6.70	3	10.0	14	11.7	

Ethnicity	Azari	12	40.0	11	36.7	21	40.0	11	36.7	46	38.3	0.267
	Perian	5	16.7	13	43.3	7	46.7	10	33.3	42	35.0	
	Kurdish	7	23.3	4	13.3	2	10.0	5	16.7	19	15.8	
	Others	6	20.0	2	6.7	1	3.3	4	13.3	13	10.8	

Income per month	<50 million rial	20	66.7	19	63.3	17	56.7	19	63.3	75	62.5	0.879
	50–100 million rial	10	33.3	11	36.7	13	43.3	11	36.7	45	37.5	

Education	<12 years	5	16.7	2	6.7	1	3.3	2	6.7	10	8.3	0.095
	High school diploma	7	23.3	1	3.3	8	26.7	6	20.0	22	18.3	
	College or university	18	60.0	27	90.0	21	70.0	22	73.3	88	73.3	

Occupation	Employed	19	63.3	21	70.0	20	66.7	24	80.0	84	70.0	0.528
	Housewife	11	36.7	9	30.0	10	33.3	6	20.0	36	30.0	

Prenatal education	No	20	66.7	17	56.7	21	70.0	16	53.3	74	61.7	0.760
	Yes	10	33.3	13	43.4	9	30.0	14	46.6	46	38.4	

**Table 2:** Comparison of the mean scores before and after the psycho-education interventions across to the study groups, using a paired *t*-test, and comparison of self-efficacy in women who have undergone CS or VD.

Variables	Self-efficacy					Intention				
	Before		After		*p* value	Before	CI-95%	After	CI-95%	*p* value
	Mean ± SD	CI-95%	Mean ± SD	CI-95%		Mean ± SD		Mean ± SD		
MI group	66.10 ± 18.25	[59.38, 73.49]	80.50 ± 22.71	[71.97, 89.16]	<0.001	1.17 ± 0.379	[1.03, 1.31]	1.57 ± 0.504	[1.38, 1.75]	<0.001
IMB group	86.87 ± 24.54	[77.70, 96.03]	103.23 ± 24.94	[93.92, 112.55]	<0.001	1.33 ± 0.479	[1.15, 1.51]	1.73 ± 0.450	[1.57, 1.90]	<0.001
IMB-App group	77.17 ± 38.67	[62.72, 91.60]	99.77 ± 30.71	[88.27, 111.20]	<0.001	1.10 ± 0.305	[0.99, 1.21]	1.70 ± 0.466	[1.53, 1.87]	<0.001
Control group	81.77 ± 40.82	[64.80, 96.60]	84.33 ± 3.67	[70.85, 97.32]	0.476	1.30 ± 0.466	[1.13, 1.47]	1.27 ± 0.450	[1.10, 1.43]	0.326

Independent-samples *t*-test: compare self-efficacy in CS and VD groups before and after the interventions										
Mode of delivery	Before		After		*p* value					
	Mean ± SD		Mean ± SD							
VD	89.04 ± 34.28		101.09 ± 27.53		*t* = 2.978, *p* = 0.004					
CS	66.30 ± 04.71		86.41 ± 30.62		*t* = 2.637, *p* = 0.009					

Mean values were significantly different from those before the intervention (paired-samples *t*-tests).

**Table 3:** Comparison of women's preferred mode of delivery by group and a comparison of women's preferred mode of delivery after the intervention to the actual mode of delivery.

Variables		Women's intention on mode of delivery			
		Before		After	
		*N*	%	*N*	%
MI group	VD	5	16.7	17	56.7
	CS	25	83.3	13	43.3

IMB group	VD	10	33.3	22	73.3
	CS	20	66.7	8	26.7

IMB-App group	VD	3	10.0	21	70.0
	CS	27	90.0	9	30.0

Control group	VD	9	30.0	8	26.7
	CS	21	70.0	22	73.3
*p* value		*p*=0.100, *t* = 6.260		*p*=0.001, *t* = 16.561	

Comparison between women's intention after the intervention and mode of delivery					
Variables		*N*		%	

Women's intention on mode of delivery after the intervention	VD	68		56.7	
	CS	52		43.3	

Delivery mode (actual birth)	VD	45		37.5	
	CS	75		62.5	

**Table 4:** Women's final mode of delivery among intervention and control groups.

Group	Mode of delivery				Total
	VD		CS		
	*N*	%	*N*	%	
MI	13	43.3	17	56.7	30
IMB	10	33.3	20	66.7	30
IMB-App	17	56.7	13	43.3	30
Control	5	16.7	25	83.3	30
Total	45	37.5	75	62.5	120
Chi-square (*χ*^2^)			*p* value =0.012		

## Data Availability

The data that support the findings of this study are available from the corresponding author upon reasonable request.

## Additional Points

*Clinical Trial Registration*. Trial registration number: IRCT20151208025431N7, Date of registration: 12/08/2018. *Transparency Declaration*. The lead author∗ affirms that this manuscript is an honest, accurate, and transparent account of the study being reported; that no important aspects of the study have been omitted; and that any discrepancies from the study as planned (and, if relevant, registered) have been explained.

## Supplementary Materials

Supplementary Materials Table S1: the contents of the intervention's sessions.
