# Supplementary material for: The Effect of “Motivational Interviewing” and “Information, Motivation, and Behavioral Skills Model” Counseling Interventions on the Choice of Delivery Mode in Pregnant Women Using Face-to-Face Training vs. Mobile App: A Randomized Controlled Trial
Source: Obstet Gynecol Int. 2024 Sep 30;2024:3071183. doi: 10.1155/2024/3071183 (PMC11458305; doi:10.1155/2024/3071183)
Supplement: Supplementary Materials — Table S1: the contents of the intervention's sessions. [file 3071183.f1.docx]

Table S1: The contents of the intervention’s sessions

| **Training session** | **MI group** | **IMB group** | **IMB-App group** | **Control group** |
| --- | --- | --- | --- | --- |
| First session | **Familiarization**: Introduction, norms, and group process, facilitating, the practice of dimensional effect of behavior, and the practice of changing assessment.  **Emotions**: Identifying emotions, exercising, and completing practice dimensions of influence with emotional dimensions and homework | **Information**: introducing and explaining the program, norms, and standards of the group interactions, asking questions to start the discussion about CS and VD, risks associated with CS, consequences of unnecessary CS, benefits of the VD | Same as IMB group | Usual antenatal care |
| Second session | **Pros and cons of the CS and VD**: brainstorming on short-term and long-term side effects and benefits of CS, comparing the pros and cons of CS and VD, describing and practicing facts and myths about the VD | **Motivation:** asking open-ended questions about unnecessary CS, providing personal feedback, reasons for needing to change the current behaviour, feedback, and affirmation; affirming desirable behavior; reflective listening, and empowering with regard to changing behaviour; summarizing; and discussing. | Same as IMB group | Usual antenatal care |
| Third session | **Values, Perspectives, and Final Assessment:** Defining values, identification and prioritization of values, adaptation of values and modes of delivery, summarizing key points of the previous sessions in perspective practice training in preparation for starting a behaviour change program (decision about VD). | **Behavioural Skills:** How to cope with VD pain, make rational decisions about choosing your mode of delivery, improve your behaviours to improve VD-related problems, conclusions. | Same as IMB group | Usual antenatal care |
